# Intercepting Premalignant, Preinvasive Breast Lesions Through Vaccination

**DOI:** 10.3389/fimmu.2021.786286

**Published:** 2021-11-24

**Authors:** Nadia Nocera Zachariah, Amrita Basu, Namrata Gautam, Ganesan Ramamoorthi, Krithika N. Kodumudi, Nagi B. Kumar, Loretta Loftus, Brian J. Czerniecki

**Affiliations:** ^1^ Department of Breast Surgery, H. Lee Moffitt Cancer Center, Tampa, FL, United States; ^2^ Clinical Science Division, H. Lee Moffitt Cancer Center, Tampa, FL, United States; ^3^ Department of Breast Oncology, H. Lee Moffitt Cancer Center, Tampa, FL, United States

**Keywords:** breast cancer, dendritic cell, vaccine, immunosurveillance, DCIS, ADH, LCIS, tumor-associated antigen

## Abstract

Breast cancer (BC) prevention remains the ultimate cost-effective method to reduce the global burden of invasive breast cancer (IBC). To date, surgery and chemoprevention remain the main risk-reducing modalities for those with hereditary cancer syndromes, as well as high-risk non-hereditary breast lesions such as ADH, ALH, or LCIS. Ductal carcinoma *in situ* (DCIS) is a preinvasive malignant lesion of the breast that closely mirrors IBC and, if left untreated, develops into IBC in up to 50% of lesions. Certain high-risk patients with DCIS may have a 25% risk of developing recurrent DCIS or IBC, even after surgical resection. The development of breast cancer elicits a strong immune response, which brings to prominence the numerous advantages associated with immune-based cancer prevention over drug-based chemoprevention, supported by the success of dendritic cell vaccines targeting HER2-expressing BC. Vaccination against BC to prevent or interrupt the process of BC development remains elusive but is a viable option. Vaccination to intercept preinvasive or premalignant breast conditions may be possible by interrupting the expression pattern of various oncodrivers. Growth factors may also function as potential immune targets to prevent breast cancer progression. Furthermore, neoantigens also serve as effective targets for interception by virtue of strong immunogenicity. It is noteworthy that the immune response also needs to be strong enough to result in target lesion elimination to avoid immunoediting as it may occur in IBC arising from DCIS. Overall, if the issue of vaccine targets can be solved by interrupting premalignant lesions, there is a potential to prevent the development of IBC.

## The Clinical Challenge of Breast Cancer Prevention

Breast cancer has become the world’s most prevalent cancer, with over 7.8 million women alive by the end of 2020 who had been diagnosed with BC in the past 5 years (1). This disease places an immense burden on society in terms of cost of medical care for these patients. As such, there is an intense effort by healthcare professionals to promote BC prevention and risk reduction.

Although clinicians are currently unable to determine which patients will develop breast cancer, they can identify patients who harbor increased risk and offer them risk-reduction options for BC prevention. Several risk calculators are available, such as the Gail Model and Tyrer-Cuzick Model, which are based on several factors within the patient’s history and characteristics such as family history, history of breast biopsies, or history of benign proliferative lesions such as atypical ductal hyperplasia (ADH) or lobular carcinoma *in situ* (LCIS). Other high-risk patients are those with hereditary cancer syndromes such as Cowden Syndrome or BRCA1/2 mutations, which are discovered by genetic testing.

Presently, the main forms of BC prevention or risk reduction are lifestyle modifications, surgery, and chemoprevention. Surgical intervention for BC prevention includes risk-reducing prophylactic mastectomy. This tends to be applied to women in whom a contralateral mastectomy is performed synchronous with the treatment of a primary tumor, or as a bilateral procedure in women at high risk of BC. In the average patient, prophylactic mastectomy reduces the risk of contralateral BC by 90–97% (2). Chemoprevention consists of selective estrogen receptor modulators (SERM) or aromatase inhibitors (AI). These are prescribed to women at high risk of BC, and this risk-reducing modality decreases breast cancer development by over 50% (3).

While surgery and chemoprophylaxis remain viable options for BC prevention, these portend high burdens for patients due to side effects of medication, potential complications of surgery, and the additional costs of these treatments. An alternative method of BC prevention that is devoid of these high costs may be vaccination. BC vaccines are an emerging therapy that utilizes the host immune system to provide protection from BC or allow interception of high-risk lesion progression to BC, while sparing patients the high burden of traditional risk-prevention strategies.

This review aims to describe our current understanding of immune response in preinvasive breast lesions and potential targets for developing therapeutic vaccination that can prevent development into invasive disease in breast cancer. For this purpose, we have reviewed the literature including primary research and review articles published in the past 10 years, focusing on the following keywords on PubMed: breast cancer, vaccine, prevention, immunosurveillance, DCIS, dendritic cell, flat epithelial atypia, atypical lobular hyperplasia, atypical ductal hyperplasia, lobular carcinoma *in situ*, and neoantigens.

## Immune Response and the Development of Breast Cancer

The breast is a complex organ, which, due to its connection to the outside world, has a multifaceted and complex immune environment. Normal breast tissue contains uniform immune cell infiltrates composed of helper T cells (CD4+), cytotoxic T cells (CD8+), B cells, and natural killer (NK) cells (4). In breast lobules, there are dendritic cells (DCs) as well as cytotoxic T cells that are uniformly present and are in close association with the breast epithelium (4). The presence of CD8+ T cells and DCs suggests a built-in defense system by antigen presentation and immune effector function. These cells within the breast parenchyma also aid in development, lactation, and involution of breast tissue (5). More importantly, they may not only play a role in development of the breast and its microbial defense, but they may also contribute a critical role in cancer immunosurveillance.

Cancer immunosurveillance is a process by which the host’s immune cells recognize and eliminate evolving tumor cells. This locally controlled inflammation may control tumor proliferation. A high density of CD8+ T cells in a tumor and nearby stroma has been associated with an improved prognosis in BC, indicating that immune effector cells have effectively identified the malignant cells and have subsequently mounted an immune response (6). A helper T cell response is activated *via* IFN-γ production, or there is direct elimination *via* cytotoxic granules (6).

Supporting cancer immunosurveillance is the observation that immunosuppression increases the risk of cancer, including BC. This has been evidenced in patients on chronic immunosuppressive medication, such as transplant recipients (7). Individuals with severe deficits of immunity have a higher likelihood of developing a variety of cancers (8).

Interestingly, chronic inflammation has been associated with cancer development (9). In chronic inflammation, myeloid suppressor cells, Th2 CD4+ T cells, and regulatory T cells work to repress CD8+ toxicity and induce pro-tumoral polarization of innate immune response *via* cytokine secretion and transforming growth factor beta (TGF-β). These polarized cells then provide a rich pro-tumoral microenvironment (10). Hence, the immune system clearly plays a role in the evolution of cancer by both promoting and preventing it.

## Precursor Lesions to Breast Cancer

The favored model of BC evolution includes a stepwise progression of early, definable precursor lesions with cellular atypia to carcinoma *in situ* to invasive breast cancer (IBC) (11). Benign proliferative lesions such as atypical ductal hyperplasia (ADH), atypical lobular hyperplasia (ALH), and flat epithelial atypia (FEA) are all considered non-obligate precursors to BC (12). Genetic studies on these lesions have shown changes in steroid hormone receptor expression levels and epigenetic changes that have been implicated as early carcinogenic events (13). The high expression of hormonal receptors such as estrogen receptors (ER) and progesterone receptors (PR) have been noted in early precursor lesions compared to the normal breast epithelial cells. This change is considered as an important influencer to develop low-grade BC (14). These features of these early precursor lesions fit into the concept of a low-grade cancer pathway, particularly since they also share histological features such as low-grade nuclear atypia (11).

### Flat Epithelial Atypia

FEA is identified as a lesion showing architectural features of columnar cell metaplasia and columnar cell changes with low-grade nuclear atypia. In addition, elongated hyperchromatic nuclei with prominent stratification can also be identified in few groups of patients with FEA (15). FEA is an uncommon premalignant lesion with 2.4% incidence rate and not independently associated with long-term BC risk (16). Studies have observed that FEA is a precursor lesion for the development of low-grade tubular carcinomas and can also upgrade into ductal carcinoma *in situ* (DCIS) (17).

### Atypical Lobular Hyperplasia

ALH is categorized as a premalignant lesion and has a high risk for development to BC. Pre- and perimenopausal status among ages 46–55 with ALH is considered as a high risk factor for development of BC compared to a postmenopausal cohort (18). ALH is usually asymptomatic and may be identified by breast imaging or found in association with other features such as radial scars, fibroadenomas, intraductal papillomas, pleomorphic LCIS, or DCIS (11).

ALH and LCIS have similar morphological findings and have been termed as lobular neoplasia. However, ALH primarily differs from LCIS based on the filling of the lobular unit and proliferation degree (19). Lesions such as ALH and LCIS are regarded as both a risk factor as well as a non-obligatory precursor for invasive carcinoma. ALH and LCIS tend to be discovered as incidental findings on core needle biopsy, as they do not have reliable imaging features attributable to them (20). “Upgrade” rate of these lesions is less than 10% (11), and surgical excision is also recommended.

### Lobular Carcinoma *In Situ*

LCIS exhibits similar histological features of ALH, but it is more proliferative compared to ALH. LCIS has about 15% risk factor for invasive BC development and may also be affected by menopausal status (21). LCIS can be detected by core needle biopsy, but it is difficult to find using breast imaging. In many cases, careful observation may be recommended to monitor signs of invasive BC progression. This includes breast self-exams, clinical breast exams, mammogram, and MRI (22). Due to the low incidence rate and lack of clear identification by breast imaging, the management of LCIS is a controversial issue (23). Surgical excision may not be required for all LCIS, but bilateral prophylactic mastectomy can be used in some patients with more aggressive form of LCIS in contralateral breast (24). Studies have shown high expressions of hormonal receptors ER and PR in LCIS patients, and these patients may largely benefit by addition of hormonal therapy (25).

### Atypical Ductal Hyperplasia

ADH is considered an immediate precursor to DCIS based on clinical and morphologic similarities between the lesions, as well as a high degree of genomic similarity with almost identical kinds of chromosomal imbalances (11). However, the prognostic differences between ADH and DCIS indicate that ADH is not just a low-grade DCIS but is actually a closely related precursor lesion. Clinically, ADH is usually associated with suspicious calcifications found on breast imaging and subsequently recommended for core needle biopsy. Once a lesion is diagnosed as ADH, surgical excision is recommended due to the “upgrade” rate of 10–20% to DCIS or invasive carcinoma (26). Many “upgraded” lesions are actually minimally sampled lesions composed of the upgraded lesion type.

ADH exhibits distinguished features of terminal ductal-lobular partial involvement with architectural disturbances, such as micropapillae and rigid bridges (27). ADH lesions are small and focal with measurement of less than 2–3 mm. With the help of the basal cytokeratin 5/6 expression detection, ADH can be pathologically distinguished from usual ductal hyperplasia (28). The genomic observation studies have supported chromosomal imbalances including deletion of chromosome 16q and 17p and gain on chromosome 1q in patients with ADH. Notably, cancer progresses from premalignant lesion on the same breast that was initially diagnosed for ADH. Menopausal status is also considered as a high-risk factor for progression of invasive BC in patients with ADH (29).

### Ductal Carcinoma *In Situ*

DCIS is a preinvasive breast lesion detected with mammography and can either present symptomatically or asymptomatically. It was reported that DCIS accounts for up to 30% of breast lesions detected by breast imaging (30). DCIS is defined as an uncontrolled proliferation of epithelial cells with the breast parenchymal structures and no evidence for the presence of invasion across the basement membrane. With the help of immunohistochemistry, DCIS may be confirmed for basement membrane type IV collagen laminin expression or presence of myoepithelial cells (31). Risk factors for DCIS development include increasing age, postmenopausal status, family history of BC, first pregnancy over 30 years of age, and hormone replacement therapy (32).

Negative ER/PR DCIS with a more aggressive phenotype was found to display increased progression to invasive BC (33). High-grade DCIS has been observed to display different molecular characteristic features compared to low-grade precursor lesions. These changes also include high expression of HER2 gene and various mutations in p53 gene (34). The treatment options for DCIS include mastectomy of the affected breast, breast-conserving surgery with or without adjuvant radiotherapy, and hormonal therapy (SERM or AI). Additionally, patients with HER2-positive DCIS may benefit from HER2-targeted therapies (35, 36).

## Rationale for Targeting Oncodrivers in Breast Cancer

The rationale for targeting oncodrivers in developing BC therapy stems from the discovery of oncogene addiction in cancer cells. Oncogene addiction, as defined by Bernard Weinstein, is the dependence of tumor cells on prolonged activity of oncodrivers for their survival and malignant phenotype (37). Such oncogene addiction offers oncodrivers as a promising target for developing cellular immunotherapy that can leverage the overexpression of oncodrivers on tumor cells to educate the immune system to detect and destroy cancer cells specifically, while avoiding adverse consequences in healthy cells. Genetically engineered mouse models of human cancer, mechanistic studies in human cancer cell lines, and clinical trials involving specific molecular targeted agents have bolstered the benefits of targeting oncodrivers for therapy development (38). While BCR-ABL in chronic myeloid leukemia was the first concrete example of an addictive oncodriver in human cancer, multiple oncodrivers have been identified in various cancers since then. Use of vemurafenib, dabrafenib in BRAF-mutated melanoma; gefitinib, erlotinib in EGFR-mutant NSCLC and crizotinib in ALK-mutated NSCLC; cetuximab, panitumumab in EGFR-amplified colorectal cancer; or tamoxifen, letrozole, and fulvestrant in ER+ BC (38, 39) have revolutionized the therapeutic outcome in patients, consolidating the rationale for oncodriver targeting.

Support for developing oncodriver-targeted therapy in BC comes primarily from the studies on HER2/Erbb2 oncodrivers. HER2 overexpression and constitutive downstream signaling in HER2+ BC cells have been identified as a poor prognostic marker that correlated with enhanced cellular proliferation and therapy resistance, invasiveness, and metastasis, leading to poor survival outcome in BC patients (40–42). Targeted inhibition of HER2 by trastuzumab, pertuzumab, T-DM1 and other strategies have revolutionized the treatment outcome for patients, further highlighting the potential for therapeutic targeting of oncodrivers (36). However, gradual development of therapeutic resistance to HER2-targeted agents in BC suggests the need for developing combinatorial strategies targeting the oncodriver, such as combining antibody-mediated inhibition and stimulation of HER2-specific immune response by DC vaccination.

Multiple models have been proposed to elucidate how targeting oncodriver addiction in cancer cells can be beneficial for targeted therapy development—namely, genetic streamlining (dismissal of non-essential cellular pathways leading to lack of compensatory signals, resulting in collapse of the cellular fitness upon abrogation of dominant signals), oncogenic shock (blockade of addictive oncoproteins subverts the balance of pro-survival and pro-apoptotic signals in favor of cell death), and synthetic lethality (inactivation of two separate pathways result in a synergistic loss of common downstream effector function and subsequent apoptosis or cell cycle arrest) (38). The outcomes of oncodriver targeting in oncogene-addicted cells (apoptosis, senescence, cell cycle arrest) are heavily context-dependent, and the signaling framework underlying such outcomes requires further research for a comprehensive understanding.

## Targeting Oncodrivers in Early-Stage Breast Cancer

Oncodrivers are proteins overexpressed in tumor cells, essential for proliferation, survival, and malignancy of cancer cells (43). While in healthy breast tissue, oncodriver proteins participate in numerous cellular events during different stages of puberty, pregnancy, lactation, and normal breast development (44), their overexpression and hyperactivity have been linked to progression and poor outcome in BC.

Perhaps the most prominent oncodriver investigated in BC is human epidermal growth factor receptor 2/receptor tyrosine-protein kinase erbB-2 (HER2/Erbb2). DCIS overexpressing HER2 has a higher propensity of progressing into invasive disease than HER2-negative DCIS (45). While HER2 overexpression is noted in more than 50% of DCIS, only 20–30% of IBC overexpress HER2 (45), suggesting a possible emergence of HER2-negative tumor cells due to immunoediting after elimination of HER2-positive cells. Combined HER2+/Ki67+ profile in DCIS has been identified as an independent predictor of local recurrence by multivariate analysis in a cohort of 868 patients (34). Overexpression of HER2 in IBC has been correlated with locally advanced stage disease, early metastasis, chemotherapy resistance, and poor recurrence-free survival in patients (46, 47).

Other members of the ERBB family of growth receptors have also been identified as oncodrivers across BC subtypes. HER3/Erbb3 is the most potent binding partner of HER2 that activates downstream signaling cascades, specifically PI3K/AKT, that contribute to cellular proliferation and survival. Therefore, HER3 hyperactivity has been associated with trastuzumab resistance in HER2-positive BC (48) and tamoxifen resistance in ER-positive BC (49, 50). In TNBC patients, overexpression of HER3 has been identified as a prognostic marker of poor 5-year DFS and 10-year OS (51–53).

EGFR/HER1 is another oncodriver protein overexpressed across BC subtypes, with more frequent appearance in IBC and TNBC subtypes. EGFR overexpression has been associated with larger tumor size and poor clinical outcome (54–56). Combined HER3-EGFR score in a cohort of 510 TNBC patients was a more comprehensive prognostic marker of worse BC-specific and distant metastasis-free survival, than individual oncodriver scores (57). Although EGFR gene amplification is rare in BC, high EGFR gene copy number predicts poor outcome in TNBC (58).

Hepatocyte growth factor receptor/receptor tyrosine kinase MET (HGFR/MET) is another oncodriver known to be overexpressed in TNBC and has been identified as an independent prognostic marker for recurrence and shorter survival (59). We have previously reported expression of HER3 and c-MET in BRCA1- and BRCA2-associated DCIS as well (60). Molecular cross-talk and downstream convergence between MET and ERBB receptor signaling has been predicted to contribute to resistance against HER2- and EGFR-targeted therapies (61).

## Targeting Breast-Specific Tumor-Associated Antigens

Tumor-associated antigens (TAAs) are the epitopes displayed on tumor cell surface and also presented by the HLAs on the surface of non-malignant cells that can be identified by comparing transcriptome of the malignant and healthy tissues and present a promising yet challenging target for therapy development due to immunogenic tolerance and lack of specificity (62, 63). While HER2 is perhaps the most widely investigated TAA identified in BC, other TAAs, e.g., MUC1, mammaglobin-A, lactalbumin, NY-ESO-1, MAGE, and MART-1, have garnered interest as potential therapeutic targets and have been reviewed comprehensively before (Criscitiello, 2012 #12430). Here, we briefly discuss the current status of therapeutic research in BC centered around some of these TAAs.

### Mucin-1

Mucin 1 (MUC1) is a transmembrane dimeric mucin, with an aberrant overexpression in over 90% BC compared to normal ductal epithelial cells of the breast tissue. Overexpression of MUC1 results from gene amplification, miRNA regulation, as well as in response to EGF or heregulin stimulation and activation of PI3K/AKT signaling (64). MUC1 has also been identified to complex with HER2, HER3, and HER4 in BC cells and mouse mammary glands. MUC1-based subunit vaccines, DNA vaccines, viral vector vaccines, DC vaccines, and glycopeptide vaccines are currently being tested (65). Subunit vaccine with MUC1 tandem repeats and MBP/BCG adjuvant induced Th1 immune response (66), activation of NK cells, and MUC1-specific CTL in mouse models of melanoma and lung carcinoma (67). In human, adjuvanted MUC1 subunit vaccine was less immunogenic in late-stage cancer patients than in early-stage patients. In metastatic BC patients, 16-amino-acid MUC1 peptide coupled to keyhole limpet hemocyanin plus DETOX adjuvant demonstrated class-I restricted CTL activation (68). Tecemotide, a VNTR MUC1 peptide delivered *via* a liposomal system, showed significantly improved survival after chemoradiation in phase II and III NSCLC trials (69). DC vaccine pulsed with survivin and MUC1 was well tolerated and showed modest antitumor immune response in NSCLC patients (70). However, L-BLP25 (Stimuvax), a liposome-based vaccine with MUC1-N terminal repeats, failed to improve overall survival in phase III trial for unresectable stage III NSCLC (71, 72). PANVAC vaccines containing transgenes for MUC1, CEA, and three T-cell costimulatory molecules (B7.1, LFA-3, and ICAM-1) have also been tested in a clinical trial with metastatic BC patients (73).

### Mammaglobin

Mammaglobin-1 (SCGB2A2) is a mammary-specific glycoprotein member of the uteroglobin family and is considered a potential diagnostic and prognostic marker for BC (74, 75). Peripheral blood and tumor tissue from DCIS and IBC patients analyzed by RT-PCR identified mammaglobin expression as the most specific molecular marker for hematological dissemination of BC cells, compared to EGFR and cytokeratin 19 (76). Mammaglobin protein and mRNA expression have been detected in 75–80% of primary and metastatic BC, as well as in bone micrometastases of BC (77). Abundance of this marker in tumor tissue and inherent immunogenicity make mammaglobin a promising target for therapy development.

### Lactalbumin

α-lactalbumin is a breast-specific differentiation protein that comprises 25% of total protein found in breast milk, overexpressed in mammary epithelial cells during lactation and in a majority of BCs, specially TNBC. Immunization of female SWXJ mice with recombinant mouse α-lactalbumin has shown dose-dependent proliferation in recall responses in the lymph node, presenting lactalbumin as a targetable TAA. A proinflammatory phenotype involving both CD4+ and CD8+ T cells and high production of IFN-γ and IL-2 was noted (78). Following this, Tuohy et al. showed presence of a proinflammatory T cell repertoire in adult women that can respond to recombinant hα-lactalbumin (79). An open-label, early phase I dose-escalation trial to test α-lactalbumin vaccine in non-metastatic TNBC patients is currently ongoing (NCT04674306).

## Targeting Neoantigens

While TAAs are “self-proteins” shared between malignant and healthy tissues, neoantigens are tumor-specific antigens (TSA), unique non-autologous proteins expressed in tumor, often derived by somatic DNA alterations such as non-synonymous point mutations, insertion/deletion, gene fusion, and frameshift mutations acquired during the tumorigenesis process, due to rapid proliferation and genomic instability (80, 81). Various aspects of neoantigen development and potential as therapeutic target have recently been extensively reviewed by Benvenuto et al. (62). Higher immunogenicity of neoantigens arising from mutations, strong tumor specificity, reduced risk of autoimmunity as foreign antigens, and protection from central immunological tolerance present neoantigens as a more favorable target than TAAs for immunotherapy development (81). Therefore, it is possible that in the TME, lower immunogenicity and weak antigen load of TAAs may require a dramatic shift in up- or downregulation of both anti- and pro-tumorigenic signals, respectively, while higher immunogenicity and abundance of neoantigens may tip the balance in favor of antitumor immune response more comprehensively by modulating only one side of the balance (47).

Multiple preclinical studies of melanoma, lung, breast, and colon cancers have demonstrated tumor rejection by neoantigen-specific vaccination, where most of the epitopes were detected by CD4+ T cells (47, 82). Zhang et al. reported identification of neoantigens from an LL2 murine lung carcinoma model by whole-exon and transcriptome sequencing of the tumor RNA. Vaccination with neoantigen-pulsed DC in mice demonstrated a stronger antigen-specific lymphocyte response, increased number of TILs including CD8+ and CD8+IFN-γ+ T cells, and inhibited tumor growth, compared to the neoantigen-adjuvant vaccination. Combination of local radiotherapy with an RNA-LPX vaccine encoding CD4+ T cell-recognized neoantigens in a CT26 mouse model resulted in activation and long-lasting memory recall response by CD8+ T cells with increased IFN-γ secretion and follow-up with anti-CTLA4 antibody resulted in complete remission of tumors (83). Higher predicted neoantigen load has been correlated with increased TIL infiltration and improved survival in melanoma, colorectal, and ovarian cancer patients receiving immune checkpoint therapy (84, 85). Immunotherapies targeting neoantigens by synthetic long peptide vaccine, DNA, RNA, and DC vaccines, and adaptive T cell therapy are currently being tested in various preclinical and clinical trials.

Owing to the low mutational burden in BC, TAAs were the primary focus of therapeutic targeting for a long time, and translational research focusing on breast neoantigens has only recently gained traction. BC is known to have a higher proportion of INDEL mutations, and TNBC is characterized by a higher number of neoantigens due to frameshift mutations, with an even higher load in BRCA-1 mutated TNBC. However, no correlation between TNBC and TIL number has been identified in the analysis of a specific cohort of TNBC patients compared with other invasive BC subtypes (86).

Building an array with non-overlapping frameshift neoantigen peptides and vaccination with reactive peptides resulted in slower tumor growth and antibody production that correlated with diminished tumor volume in 4T1 murine model (87). In another study, PALB2, ROBO3, PTPRS, and ZDHHC16 were identified as neoantigens in advanced BC patients. Whole tumor exome analysis from the PDX mouse models generated from those patients identified a large number of non-synonymous single nucleotide variants. Following determination of predicted HLA binding affinity and functional evaluation by ELISPOT, neoantigen-specific T cells were shown to inhibit patient tumor growth implanted in NSG immunodeficient mice (88). Whole exome and RNA sequencing from BC tissues and neoantigen prediction among exonic mutations showed positive correlation between neoantigen load and non-synonymous single-nucleotide variations (nsSNVs). Using primary tumor cells established from pleural fluid of a BC patient, co-culturing neoantigen-pulsed DCs with autologous peripheral lymphocytes resulted in induction of CTLs *ex vivo* (89). Evaluation of the neoepitope burden in BC from TCGA using a predictive algorithm called EpitopeHunter showed that total mutational burden was highest for TNBC, followed by HER2+ BC and lowest for ER+/PR+/HER2− BC and the neoepitope load correlated with such mutational burden (90). Liu et al. have identified >700 non-silent somatic variants in BC patients obtained from the cBioportal dataset and observed higher single-nucleotide variant neoantigens in the elder population (>60 yrs) and identified multiple high-frequency mutations in PIK3CA and AKT that can be recognized by various HLA molecules (91). On the other hand, mutations in the ESR1 gene coding for the ER protein has been identified to be relatively common in metastatic, therapy-resistant cancers and contribute to shorter progression-free survival in endocrine BC (92, 93) and, hence, can be employed for developing neoantigen-pulsed DC vaccination for ER-positive BC.

Identification of neoantigens that are “private” antigens specific for individual patients requires a long and arduous bioinformatic screening followed by experimental validation to verify the epitopes, as well as both quality (specificity and affinity of infiltrating immune cells towards neoantigens) and quantity (number of activated TIL). This can become a limiting factor towards successful development of neoantigen-specific immunotherapy. However, research in the past few years has made remarkable progress towards that direction (94). In patients with NSCLC (95) and melanoma (96), personalized neoantigen-pulsed DC vaccination was found to be safe, reliable, and beneficial to reduce tumor burden and metastatic lesions. Neoantigen targeting with synthetic long peptide or polyepitope DNA vaccines in 4T1 and E0771 murine mammary carcinoma models have led to initiation of two clinical trials (NCT02427581 and NCT02348320) enrolling TNBC patients to test safety of personalized neoantigen vaccines using the same platforms (81). In a recently completed phase I/II clinical trial in TNBC patients, 40% of the patients showed pathologic complete response after receiving cyclin B1/WT-1/CEF tumor antigen-loaded DC vaccination with preoperative chemotherapy (NCT02018458). As summarized by Benvenuto et al. (62) and Han et al. (97), multiple clinical trials are currently testing safety, immunogenicity, effects on pathological complete response, TIL percentage, recurrence rate and survival in TNBC, BRCA-mutated and other subtypes of BC.

## Drug-Based Chemoprevention *vs.* Immune-Based Prevention

Patients with a diagnosis of any of the known precursor lesions are considered to be at significantly increased lifetime risk of developing BC, with estimated 10-year cancer risks of 17.3% with ADH, 20.7% with ALH, 23.7% with LCIS, and 26.0% with severe ADH (98). Because of this increased risk, these women are closely surveilled, and chemoprevention is recommended. One major analysis of women with benign proliferative lesions in four chemoprevention trials (NSABP P-1, MAP.3, IBIS-I, and IBIS-II) has demonstrated that chemoprevention with endocrine therapy was associated with 41–79% relative risk reduction of BC (3).

Despite this high rate of risk reduction, there is underutilization and low adherence of chemoprevention by high-risk patients (99). Endocrine therapy places a high burden of side effects on the patient, including major risk of venous thromboembolism, stroke, osteoporosis, and endometrial cancer. More common and noticeable side effects patients may experience include menopause-like symptoms, joint aches, and mental fog. These medications are also recommended to be taken for 5 years for maximum benefit, which, for some patients, may be a significant burden.

Cancer immunoprevention modulates the immune system to recognize aberrant cells and prevent the initiation and progression to malignancy. Potential advantages to immune-based cancer prevention over drug-based chemoprevention include (1) high specificity and adaptability of immune responses (adaptive immunity is specific to a given antigen and can adjust to changes within the antigenic repertoire); (2) favorable toxicity profile (immune strategies—cancer vaccines in particular—appear non-toxic in the majority of cases); (3) ability to generate immunological memory, providing long-term (potentially lifelong) protection (not achievable with drugs); and (4) ease of administration (i.e., several vaccinations with occasional boosts *versus* daily dosing for many years with chemo preventive agents) (100). Vaccination has been proven to be a proficient and cost-effective means of eradicating many pathogens; hence, it stands to reason that vaccination may be an efficient means for immunoprevention of cancer, especially in high-risk individuals.

Vaccinations for cancers with viral etiologies are widely available and have been shown to be efficacious, such as vaccination against human papilloma virus (HPV) and hepatitis B. Since implementing these vaccination programs, the incidence of cervical and hepatic cancers have been reduced (101). However, unlike cervical and hepatic cancer, BC is a complex and multifactorial disease that does not have a target pathogen for vaccination. A preventative vaccine would rely on targeting normally overexpressed, mutated, or cancer-specific targets (102). Some targets considered in the development of BC vaccines include targeting oncodrivers, i.e., overexpressed proteins, tissue-specific antigens, and targets that are expressed in cancer tissue, but not in normal cells (102).

## CD4 T Cell Response in Cancer

**Figure 1:**
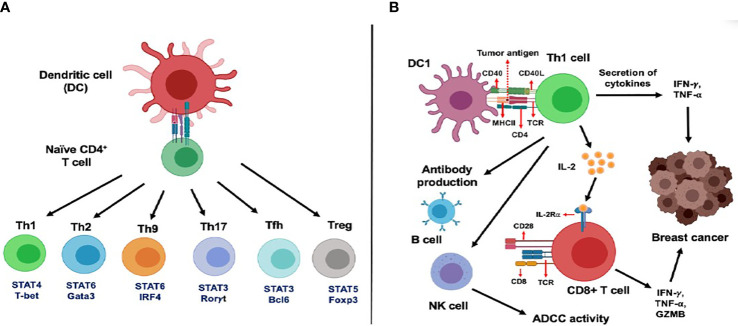
Functional subsets of CD4+ T cells and role of CD4+ T cells in the activation of CD8+ T cells in cancer. **(A)** Dendritic cells (DC) regulate differentiation and polarization of naїve CD4+ T cells into various T helper cells subsets such as Th1, Th2, Th9, Th17, Tfh and Treg cells. **(B)** DCs primed with tumor antigenic peptides can present the antigens to Th1 cells through the MHC class II molecule. Next, these activated Th1 cells secrete two important Th1 cytokines: IFN-γ and TNF-α, leading to direct tumor growth inhibitory effects, mediated by induction of apoptosis, senescence, cell cycle arrest and proliferation arrest. Secretion of IL-2 cytokine from Th1 cells is known to mediate activation and proliferation of IL-2Rα expressing CD8+ T cells which leads to enhanced anti-tumor response. In addition, Th1 cells can regulate B cell-mediated antibody production and NK cell-dependent antibody dependent cellular cytotoxicity (ADCC) in cancer.

For a long time, cancer immunotherapies have focused on CD8+ T cells as the principal adaptive immune T cell subset, known for their antitumor cytotoxic response. However, the potential of CD4+ helper T (Th) cells in tumor suppression has recently gained attention in the field of immunotherapy. The overview of various CD4+ T cells (**Figure 1**), its subsets, and their role in mediated tumor immune response has been reviewed by our group previously (https://doi.org/10.3389/fimmu.2021.669474).

## Immunoediting

Since the concept of tumor suppression by immune system introduced by Paul Ehrlich (103) and the hypothesis of cancer immunosurveillance proposed by Burnet and Thomas (104), roles of the immune system in shaping up tumorigenesis and therapeutic response have been established unequivocally. Research by Schreiber and others led to the refinement of the immunosurveillance concept to the hypothesis of cancer immunoediting (105–107) that acknowledges the complexity of tumor immune response in a far more comprehensive manner.

Schreiber et al. envisioned immunoediting in cancer as a three-step process, “the three Es of cancer immunoediting” (108), namely, Elimination, Equilibrium, and Escape. During this final step of immune escape, surviving tumor cells with genetic and epigenetic changes rendered resistant to detection and deletion by the host immune system enter the phase of uncontrolled growth and become clinically observable malignant disease (105, 109–111).

## Immune Escape Mechanisms in Breast Cancer

Mechanisms of immune escape in cancer has been reviewed extensively elsewhere (112–114). Loss of immune detection and activation (absence of strong tumor antigen, lack of DC and T cell priming, tumor antigen processing and presentation, reduced MHC class I expression, upregulation of HLA-G to promote tolerogenic phenotype), enhanced resistance to cytotoxicity and apoptosis (constitutive activation of STAT3, oncodriver proteins, e.g., HER2, HER3, EGFR, anti-apoptotic protein Bcl-2), and shaping of an immunosuppressive tumor microenvironment (TME) [secretion of immunosuppressive cytokines TGF- β, VEGF, and metabolic factors IDO, PGE-2; adaptive immune resistance by upregulation of PD1/PDL-1, LAG3, Tim3; induction of Tregs, tumor-associated macrophages (TAMs), and MDSCs] have been shown to be the potential mechanisms of immune escape in BC and many other subtypes of cancer (110, 114–116).

Inherently low immunogenicity of BC contributes to immune escape, and progression from preinvasive to invasive disease. In residual triple-negative breast cancer (TNBC) after neoadjuvant chemotherapy, Ras-MAPK, PD-L1, and TIL infiltration showed a strong correlation and increased Ras/MAPK activation correlated with a poor TIL phenotype in the residual cancer (117). Expression of PD-L1 has been shown to increase in TNBC after neoadjuvant chemotherapy, while PD-L1 amplification has been detected in triple-negative IDC but not DCIS in a separate study (118). Similarly, HER2 amplification in HER2+ DCIS and IDC has been associated with co-amplification of a nearby cytokine cluster that inversely correlates with intratumoral frequency of granzyme-secreting CD8+ T cells (116). These observations underline the clinical relevance of therapeutic strategies targeting immune escape mechanisms to amplify therapeutic impact on patient outcome.

## Loss of Anti-HER2 Th1 Response During Breast Tumorigenesis

While a basal level of anti- HER2 Th1 response is reported in healthy individuals, reflective of immune regulation by HER2, suppression of this Th1 response by malignancy breaks the immune protection, ultimately leading to progressive HER2+ tumor development (44, 119).

We have previously reported an incremental loss of Th1 immunity observed in HER2+ DCIS patients, with negligible responses in HER2+ IBC patients (119). A further sequential loss of HER2-specific Th1 response takes place in advanced IBC patients (120). Our group has also reported that restoration of the anti-HER2 Th1 response culminated in improved survival in HER2+ BC patients (46, 119). The molecular basis of this effect was investigated, and we now know that the Th1 cytokine IFN-γ increased E3 ubiquitin ligase Cullin-5, which led to ubiquitination and degradation of surface HER2 receptors, translating into tumor senescence and diminished tumor growth (121).

To augment expression of MHC-I molecules on tumors and efficient cytotoxic responses by HER2-specific CD8+ T cells, crosstalk between trastuzumab and IFN-γ and TNF is critical (122). Moreover, positive prognosis is anticipated by the presence of infiltrating Th1 IFN-γ-producing cells (123). Not only this, in patient-derived xenograft (PDX) ER− BC model, stimulation of IFN-γ/STAT1 pathway is identified as a prognostic marker of chemotherapy resistance. An ongoing clinical trial (NCT03112590) aims to elucidate how augmenting the Th1 response can be a pivotal immunotherapeutic tool, by testing a combination of IFN-γ with paclitaxel, trastuzumab, and pertuzumab in HER2+ BC. Loss of Th1 responses leading to tumor progression could point towards increase in apoptosis of CD4+ T cells *via* Fas pathway or tolerance to tumor antigens (for instance, CTLA-4 and PD-1) (124, 125). To this end, our group found that improved survival was achieved in TUBO HER2+ murine mammary carcinoma model upon delivering anti-PD1 antibody, post HER2-DC1 vaccination (126).

Similarly, our group has reported a progressive loss of Th1 immunity against HER3 in IBC patients, which was most pronounced in TNBC patients compared to the healthy donors, directing towards a fair chance to boost the Th1 responses to achieve improved survival (127).

## Role of Dendritic Cells in Th Cell Differentiation

DCs are considered as master regulators of immune system and play a critical role in activation of adaptive immune cells (128). Three types of DC subsets have been identified, namely, myeloid/conventional DC1 (cDC1), myeloid/conventional DC2 (cDC2), and plasmacytoid DCs (pDC) (129, 130). The functional status of DCs is mainly classified by high expression of MHC class I and class II molecules, and expression of various co-stimulatory receptors including CD80, CD86, CD83, CD40, leucocyte functional antigen (LFA) family of adhesion molecules, and heat stable antigen (131, 132). cDC1s are involved in various antigen presentation to CD8+ T cells and stimulate cytotoxic activity. In addition, DC subsets can also mediate differentiation of CD4+ T cells into Th1, Th2, Th9, Th17, Tfh, and Treg cells (47, 132, 133).

Secretion of IL-12 by cDC1s can mediate Th1 polarization and NK cells infiltration. Stimulation of DCs with lipopolysaccharides has been shown to induce expression of Notch ligand delta, leading to Th1 polarization (134). On the other hand, OX40 ligand activation in myeloid cDC2 can mediate Th2 polarization (135). Preferential MHC II expression and higher IL-12 secretion by cDC2 make them better equipped to contribute to CD4+ Th cell polarization than cDC1 (135). A previous study has shown that calcium signaling activation in human PBMC-derived myeloid DCs can inhibit IL-12 production, which leads to CD83+ DCs activation and regulation of Th2 differentiation (136). Stimulation of DCs by prostaglandin-E2 can facilitate CD4+ T cells into Th2 phenotype polarization (137). Activation of dectin-1 in DCs has been reported to promote Th9 polarization *via* expression of OX40L, TNFSF15, Syk, Raf1, and NF-κB signaling cascades (138, 139). In response to various cytokines such as IL-6, IL-1β, IL-23, and TGF-β, DCs also induce polarization of the Th17 phenotype (140). In addition, stimulation of DCs with prostaglandin-E2 was also identified to control the balance between IL-12 and IL-23 cytokines and promote Th17 differentiation by inhibiting Th1 and Th2 polarization (141).

Cooperation between DCs and B cells has been shown to regulate MHC class II molecule–mediated antigen presentation, which stimulates Tfh cells polarization (142). Another study has shown that induction of inducible costimulatory (ICOS) ligand expression in plasmacytoid DCs can induce Treg cells polarization (143, 144). IL-10 cytokine can negatively regulate DC functionality, expression status of MHC class II and IL-12 production, which converts DCs to promote Treg polarization (145).

## Using DC Vaccination to Target HER2

**Figure 2:**
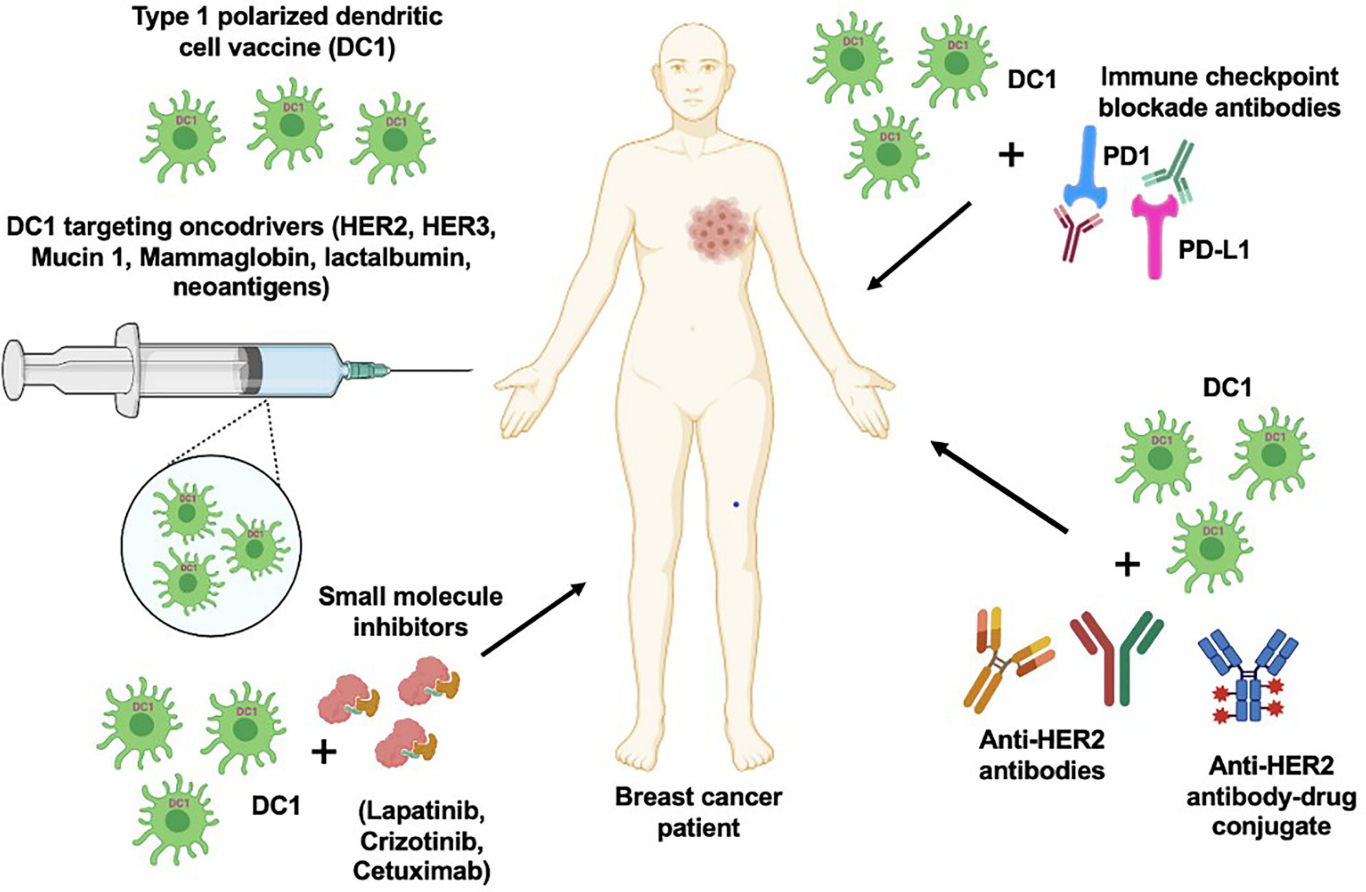
DC1 vaccine with combination therapeutic approach. DC1 vaccination targeting various oncodrivers such as HER2, HER3, mucin 1, lactalbumin and neoantigens can be an effective strategy to improve therapeutic efficacy and survival outcome in breast cancer patients. In addition, combination of DC1 vaccine with clinically available targeted therapies such as anti-HER antibodies, small molecular inhibitors and immune checkpoint blockade antibodies can also enhance the patient outcome.

DCs are used as a vaccine delivery tool to generate antitumor immune response in BC-targeting tumor antigens (146). Potential of HER2-targeted immunotherapy using the DC platform in BC has been reviewed previously (147, 148). Our group has showed type I polarized DC vaccine pulsed with HER2 peptides (HER2-DC1) generated strong anti-HER2 CD4+ T cell immunity in vaccinated HER2+ BC patients (**Figure 2** **)**, as well in ER+/HER2+ and ER−/HER2+ BC patients, resulting in improved pathologic complete response in HER2+ DCIS and early BC patients (149, 150). In addition, HER2-DC1 vaccination in combination with anti-estrogen therapy enhanced HER2-specific Th1 immunity and reduced disease recurrence, compared to HER2-DC1 monotherapy in ER+/HER2+ patients (151). This study outcome emphasized therapeutic promises of combination therapy approach for HER2/ER-positive BC patients.

CD8α DCs are one of the subtypes of DCs which display high expression of IC-type lectin cell surface receptor DEC205 and are involved in cross antigen presentation and activation of CD4+ and CD8+ T cells (152). DEC205 receptor expressing CD8α DCs pulsed with extracellular domain peptides of the HER2/neu protein was able to generate CD4+ and CD8+ immune response and B cell-mediated antibody production in preclinical model of HER2/neu+ BC. Notably, prolonged antitumor response, rejection of secondary tumor challenge, and improved survival were observed following DC vaccination in HER2/neu+ BC preclinical model (152, 153). GP2 is an MHC class I recognizing immunogenic peptide obtained from the intracellular domain of HER2 protein and has been shown to induce strong CD8+ T cell-mediated antitumor response in HER2+ BC (154). Previously, phase II clinical trial investigating the efficacy of GP2 peptide vaccination in combination with immunoadjuvant GM-CSF treatment showed CD8+ T cells activation with improved 5-year DFS in HER2+ BC patients (155). In addition, GP2 peptide pulsed DC vaccination in transgenic mice induced HER2/neu specific CTL in preclinical BC model (156).

E75 is another immunogenic peptide derived from the extracellular domain of HER2/neu protein that stimulates HER2/neu-specific CTL to cause tumor cell lysis in HER2/neu transgenic mouse model (157). E75 peptide vaccine in combination with immune stimulatory cytokine GM-CSF treatment generated CD8+ T cell immune response and improved DFS in HER2+ BC patients (158). E75 peptide pulsed DC vaccination efficacy has also been tested in clinical trials with early stage and invasive BC patients (159). A phase III clinical trial with E75 peptide vaccine in combination with GM-CSF therapy is currently ongoing in low to intermediate HER2+ BC patients (160).

HER2/neu transgene modified dendritic cell (DCneu) vaccine efficacy has been studied in HER2/neu+ BC mouse model where it suppressed Treg cell activity and enhanced Th1 immune response and HER2/neu specific humoral response. In addition, DCneu vaccination was able to induce strong tumor inhibitory effect and long-lasting antitumor response by protecting from secondary tumor re-challenge in mice (161). The CD4+ T cells recognizing epitope P30 have been reported to enhance CD8+ T cell-mediated immune response. Vaccination with DCs engineered with HER/neu oncogene and P30 epitope eliminated immunological tolerance by self-antigen and induced strong CD4+ and CD8+ T cells immune response in HER2/neu transgenic mouse model (162). Another study showed that treatment of DC vaccine pulsed with truncated neu antigen, interleukin 15 (IL-15), and IL-15Rα reduced mammary carcinoma development and inhibited HER2-dependent Akt signaling pathway in HER2/neu transgenic mice. DC vaccination was able to stimulate CD4+ Th1 immune response and eradicate HER/neu+ tumors in preclinical model (163).

## Vaccination in Addition to Targeted Therapy for HER2+ Breast Cancer

Although HER2-targeted therapies, including anti-HER2 monoclonal antibodies (mAb) such as trastuzumab and pertuzumab, have improved the pathologic complete response (pCR) and DFS, development of resistance, metastasis, and disease recurrence noted in patients remain the main obstacle (164). Notably, combination treatment of DC vaccine with trastuzumab (**Figure 2**) was able to induce strong CTL response and improve anti-HER2 Th1 immune response in HER2+ BC (6). This opened up a new avenue to enhance the efficacy of trastuzumab by combining with HER2-DC1 or immunostimulatory cytokines in HER2+ BC patients. Previous studies have shown that trastuzumab treatment in combination with DCs pulsed with HER2 peptides E75 or GP2 and GM-CSF were able to generate CD8+ T cell immune response in HER2+ BC patients and enhanced DC-mediated presentation of E75 peptides in preclinical model of HER2+ BC (158, 159, 165). In addition, clonal expansion of E75 peptide-specific CD8+ T cells after combination treatment was identified as a key benefit (166). Another study showed HER2/neu oncogene constructed DC vaccine and trastuzumab combination treatment prevented spontaneous mammary carcinoma growth in HER2/neu-overexpressing transgenic mice, as the combination treatment was able to induce strong HER2/neu-specific CD8+ CTL immunity, which prevented tumor growth in mice (126, 167). Recently, it has been observed that HER2-DC1 vaccine, in combination with anti-HER2 antibodies, was able to completely arrest tumor growth in HER2/neu BC preclinical model (121).

Pharmacological inhibition of HER2-dependent PI3K/Akt and MAPK/ERK signaling activation is another attractive therapeutic option in HER2+ BC patients. Dual targeting of HER2 signaling with trastuzumab and tyrosine kinase inhibitor lapatinib is used to treat patients with locally advanced HER2+ BC (168) and has been shown to inhibit HER2 mediated downstream signaling cascades *via* PI3K/Akt and MAPK/ERK activation in HER2+ BC (169). Addition of HER2-targeted therapies such as lapatinib and trastuzumab can further potentiate therapeutic efficacy of DC1 vaccine and overcome therapy resistance in patients. A recent study has observed that combination treatment of class I and class II peptide pulsed HER2-DC1 vaccine with Akt antagonist MK-2206 was able to control the tumor growth in HER2/neu+ BC preclinical model (170). In support of this observation, Th1 cytokine IFN-γ in combination with MK-2206 treatment displayed similar tumor inhibitory effects in HER2/neu+ BC preclinical model and various HER2+ human BC cells (170).

## Utilizing Anti-HER2 Vaccines as a Preventative Strategy

While trastuzumab and pertuzumab are effective adjuvant treatments for HER2+ BC, they are not for use in the preventative setting. Numerous attempts at various modalities for an anti-HER2 BC vaccine have been attempted in order to be used for prevention or in a neoadjuvant setting. Peptides within the HER2 protein can be recognized by CD8+ T cells in MHC class I molecules, and one protein that has been studied for this purpose is the E75 peptide. The E75 vaccine is a peptide vaccine that elicits a CD8+ CTL response. Because it only elicits a CTL response, immunization against this single peptide results in a low-level, short-lived response with paucity of activation of other components of the immune system (171).

Anti-peptide vaccination may be more effective in cancer cells with low HER2 expression because these cells exhibit high MHC class I expression and are more easily recognized by CD8+ T cells, allowing for elimination of tumor cells (46). Peptide vaccination is not likely effective in HER2-high BC due to the downregulation of MHC class I expression, which inhibits CTL recognition (46, 172). Vaccines aimed at targeting HER2-high expressing tumors should elicit activation of CD4+ T helper cells, secrete IFN-γ and TNF-α, which will upregulate expression of MHC class I, increasing sensitivity to CD8+ CTL-mediated lysis. This leads to humoral immunity and long tumor immunologic recognition.

It has been observed that healthy individuals actually harbor anti-HER2 CD4+ Th1 cells that secrete IFN-γ and TNF-α, and in individuals with HER2+ BC, this immune response is diminished (119). DC vaccines have been shown to prime an immune response in vaccinated subjects and, in one study, has achieved pCR in 18% of subjects and eradication of HER2 expression in residual DCIS in 50% of subjects without pCR (173). DCs are efficient in the presentation of antigens and signal activation and polarization of T cells into CTLs and Th cells (174, 175). DCs are also efficient in production of IL-12, which polarizes T cells toward the IFN-γ Th1 phenotype and also has antiangiogenic capabilities, activates natural killer cells, enhances adaptive immunity, and improves sensitization to tumor antigens (176). Utilizing these properties, DC vaccination against HER2 would provide long-term tumor immunity, even against tumor cells expressing high HER2 levels.

Anti-HER2 DC vaccines have seen more clinical success in early stages of BC—mainly in the DCIS phase (6, 177). This may be due to the fact that in advanced disease, DCs are unable to mount a strong enough immune response to overcome the overwhelming immunosuppressive TMEs that have escaped immunosurveillance (6). During the DCIS phase, tumor cells and the immune system have achieved a state of equilibrium. Tipping the scale in favor of tumor cells results in invasive disease, while moving the scale towards the immune system results in eradication of disease. Anti-HER2 DC vaccination eliminates equilibrium, giving the immune system the boost it needs for elimination of tumor cells.

## Conclusion

Breast cancer can be a devastating disease; therefore, a great degree of importance is placed on risk reduction and prevention. Identification of which patients would benefit from risk reduction strategies is critical; these patients include genetic mutation carriers, patients with strong family histories, personal history of breast cancer, and/or history of proliferative breast lesions. The current risk reduction and prevention strategies for high-risk patients include prophylactic mastectomy and chemoprevention, which unfortunately are not benign strategies and may pose significant burden to patients. Vaccination against breast cancer-specific oncodrivers or tumor-associated antigens shows promise in intercepting progression to IBC by boosting host immunity to recognize aberrant cells and eradicate them before development of invasive disease. Breast cancer-specific vaccination does not pose significant incumbrance to patients, may be more cost-effective, and may provide long-term protection. However, vaccination against breast cancer is not a one-size-fits-all approach and requires targeting specific antigens that may be present in one type of breast cancer and not another. Targeting benign, premalignant conditions such as ADH, FEA, or LCIS may provide means for risk reduction; however, utilizing vaccines to target a specific antigen in these conditions remains elusive. Certain types of vaccines, such as anti-HER2 DC vaccines given to patients with DCIS, have shown promise, but have yet to be studied in the preventative setting.

The current research in breast cancer vaccination has yet to scratch the surface of potential targets and has mainly focused on oncogenes and peptides such as HER2 and E75 in patients who have already been diagnosed with DCIS or breast cancer. Oncodriver expression may differ according to etiology as non-hereditary DCIS lesions express more HER2 while BRCA mutation carriers express HER3 and C-MET in DCIS, suggesting targeting a single oncodriver may not be sufficient for prevention of all DCIS. Breast-specific tumor-associated antigens such as mammaglobin, MUC1, and lactalbumin may provide a broader range of coverage in a preventative setting, but studies utilizing these proteins in targeted therapies are still in their infancy. The question that remains is, which breast cancer-specific vaccination target will provide the most effective risk reduction with broad coverage for the different subtypes of breast cancer? Moreover, another question still to be addressed is if shared neoantigens such as fusion proteins and frameshift mutations could also be effective targets because of being highly immunogenic in nature. Elucidating a clear target for future successful vaccination strategies to intercept premalignant, preinvasive breast lesions continues to be a difficult task, but eventually will provide a powerful tool for all at-risk patients.

## Data Availability Statement

The original contributions presented in the study are included in the article/supplementary material. Further inquiries can be directed to the corresponding author.

## Author Contributions

NZ, AB, NG, RG, and KK contributed to writing, editing, graphical presentation, and final approval. LL and BC contributed to editing and final approval. All authors contributed to the article and approved the submitted version.

## Funding

This work was supported by Department of Defense (Award# W81XWH-16-1-0385) and Pennies in action to Dr. Brian Czerniecki.

## Conflict of Interest

BC has a patent application filed for intellectual property on a human version of DC1. LL has stock in Johnson & Johnson, Pfizer, Eli Lilly, Gilead, and Amgen.

The remaining authors declare that the research was conducted in the absence of any commercial or financial relationships that could be construed as a potential conflict of interest.

## Publisher’s Note

All claims expressed in this article are solely those of the authors and do not necessarily represent those of their affiliated organizations, or those of the publisher, the editors and the reviewers. Any product that may be evaluated in this article, or claim that may be made by its manufacturer, is not guaranteed or endorsed by the publisher.
